# Phylogeographic mitogenomics of Atlantic cod *Gadus morhua*: Variation in and among trans‐Atlantic, trans‐Laurentian, Northern cod, and landlocked fjord populations

**DOI:** 10.1002/ece3.3873

**Published:** 2018-06-01

**Authors:** Linda A. Lait, H. Dawn Marshall, Steven M. Carr

**Affiliations:** ^1^ Genetics, Evolution, and Molecular Systematics Laboratory Department of Biology Memorial University of Newfoundland St. John's NL Canada; ^2^ Centre for Biodiversity Genomics, Department of Integrative Biology University of Guelph Guelph ON Canada

**Keywords:** bottlenecks, fish, founder effect, mitogenomics, phylogeography, Pleistocene glaciations

## Abstract

The historical phylogeography, biogeography, and ecology of Atlantic cod (*Gadus morhua*) have been impacted by cyclic Pleistocene glaciations, where drops in sea temperatures led to sequestering of water in ice sheets, emergence of continental shelves, and changes to ocean currents. High‐resolution, whole‐genome mitogenomic phylogeography can help to elucidate this history. We identified eight major haplogroups among 153 fish from 14 populations by Bayesian, parsimony, and distance methods, including one that extends the species coalescent back to ca. 330 kya. Fish from the Barents and Baltic Seas tend to occur in basal haplogroups versus more recent distribution of fish in the Northwest Atlantic. There was significant differentiation in the majority of trans‐Atlantic comparisons (Φ_ST_ = .029–.180), but little or none in pairwise comparisons within the Northwest Atlantic of individual populations (Φ_ST_ = .000–.060) or defined management stocks (Φ_ST_ = .000–.023). Monte Carlo randomization tests of population phylogeography showed significantly nonrandom trans‐Atlantic phylogeography versus absence of such structure within various partitions of trans‐Laurentian, Northern cod (NAFO 2J3KL) and other management stocks, and Flemish Cap populations. A landlocked meromictic fjord on Baffin Island comprised multiple identical or near‐identical mitogenomes in two major polyphyletic clades, and was significantly differentiated from all other populations (Φ_ST_ = .153–.340). The phylogeography supports a hypothesis of an eastern origin of genetic diversity ca. 200–250 kya, rapid expansion of a western superhaplogroup comprising four haplogroups ca. 150 kya, and recent postglacial founder populations.

## 1 INTRODUCTION

An understanding of population genetic structure, particularly in species threatened with extinction, is critical to conservation and preservation of biodiversity. Genetic structure can be influenced by a number of factors including habitat loss, changes to environmental conditions, overexploitation, and the introduction of invasive species (Frankham, 1995; Wilson, 1988). The marine environment has been particularly impacted by overexploitation and environmental change (Alheit & Hagen, 1997; Carpenter et al., 2008; Hoelzel, 1999; Lluch‐Belda et al., 1992; Schultz, Baker, Toonen, & Bowen, 2009). For example, the most recent Pleistocene glaciations (ca. 110–12 kya) disrupted ocean currents, decreased sea temperatures, and caused substantial sea level drops in the North Atlantic Ocean (Pielou, 1991; Shaw, 2006). More recently, heavy fishing pressures have caused severe population declines in many marine fish species (O'Dea & Haedrich, 2002; Rose, 2007). Unlike terrestrial and freshwater species that frequently exhibit strong population genetic structure from barriers to gene flow such as rivers, waterfalls, mountains, and deserts, marine species typically experience fewer and less formidable physical obstacles. While some species are restricted by habitat requirements or feeding habits (e.g., tropical reef fishes) and show clear population genetic structure (Shulman & Bermingham, 1995), pelagic species often have wide distributions, large population sizes, high fecundity, and extensive gene flow (Palumbi, 1992), and tend to display weak population structure in the absence of natal philopatry (Carr, Duggan, Stenson, & Marshall, 2015; Castro et al., 2007; Vis, Carr, Bowering, & Davidson, 1997; Ward, 1995).

**Figure 1:**
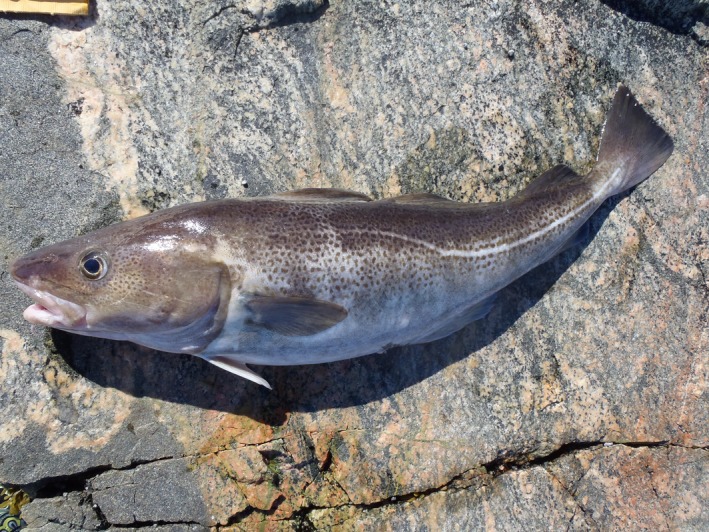
Atlantic cod (*Gadus morhua*) caught near Hopedale, Newfoundland and Labrador, Canada. Copyright: Linda Lait (2013)

**Figure 2:**
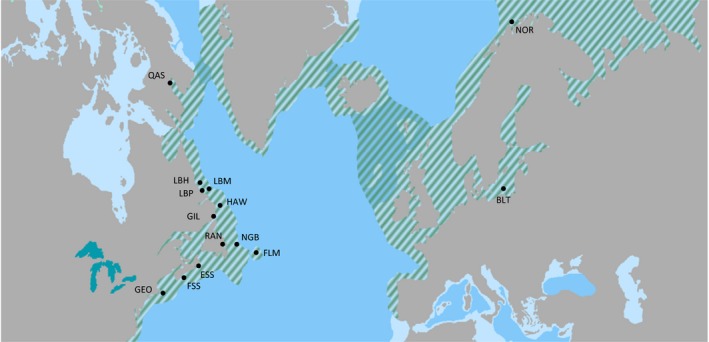
Distribution of Atlantic cod across the North Atlantic Ocean (shaded lines). Sampling locations, shown by circles, are as follows: QAS (Lake Qasigialiminiq, Nunavut), LBH (Hopedale, Labrador), LBM (Makkovik, Labrador), LBP (Postville, Labrador), HAW (Hawke Channel, Labrador), GIL (Gilbert Bay, Labrador), RAN (Random Island, Newfoundland), NGB (North Cape of the Grand Banks, Newfoundland), FLM (Flemish Cap), ESS (Eastern Scotian Shore), FSS (Fundy—Scotian Shore), GEO (Georges Bank), NOR (Norway at Tromsö), and BLT (Baltic Sea at Sopot). Figure modified from FishBase (2013)

Atlantic cod (*Gadus morhua* L. 1758; Figure 1) are demersal omnivores found along the continental shelves of the North Atlantic Ocean. Offshore populations commonly migrate in large aggregations between spawning grounds and feeding grounds to find their main food source, capelin (*Mallostus villosus*; Lear & Green, 1984; Pálsson & Thorsteinsson, 2003; Templeman, 1979). The distribution extends as far south as Cape Hatteras (North Carolina), northward through New England and Atlantic Canada, across the continental shelves of Greenland and Iceland, through the North Sea, and into the Baltic and Barents Seas (Figure 2). Of particular ecological interest are three landlocked coastal fjords on Baffin Island, Nunavut, Canada: Lake Ogac off Frobisher Bay, and Lakes Qasigialiminiq and Tariujarusiq off Cumberland Sound (Hardie, 2003). These lakes are described as meromictic, with a freshwater layer at the surface and saltwater of varying concentration underneath (Hardie, 2003; McLaren, 1967). Their populations are estimated at no more than 500–1,000 cod each. These fish survive in extremely cold and seasonally frozen waters, they mature at a much greater size and age than their marine counterparts, and they feed on echinoderms, molluscs, polychaetes, and other cod (Hardie, 2004; Hardie & Hutchings, 2011; Patriquin, 1967).

Atlantic cod previously supported one of the most important commercial fisheries in the world (Halliday & Pinhorn, 1996). Heavy fishing in the latter half of the 20th century led to severe population declines, first in Europe and then in North America. The northeast Newfoundland and Labrador stock (“Northern cod” NAFO 2J3KL) declined by >99%, and other areas by >90% (COSEWIC, 2003). As a result, a moratorium on commercial cod fishing was introduced in Canadian waters in 1992. A food fishery, “Sentinel Survey,” and some limited local commercial fishing have since been reopened (Rose, 2007). Populations in the Northwest Atlantic are categorized as Vulnerable on the IUCN Red List (International Union for Conservation of Nature; Sobel, 1996). In 1998, the Committee on the Status of Endangered Wildlife in Canada (COSEWIC) assessed all Canadian populations as of Special Concern, which was reassessed in 2003 to Endangered for the Newfoundland and Labrador populations, Threatened for the North Laurentian populations, and of Special Concern for the Maritimes and Arctic populations (COSEWIC, 2003). In 2010, the North Laurentian, South Laurentian, and Southern populations were reassessed as Endangered (COSEWIC, 2010).

Based on distinctions in meristic analysis of vertebral counts (Templeman, 1979, 1981), cod in the waters off Newfoundland and Labrador have traditionally been managed as five stock divisions designated by the North Atlantic Fisheries Organization (NAFO; see Table 1). Elsewhere, cod stocks are delineated by marine area (e.g., Baltic Sea, Barents Sea, North Sea, and Iceland) with numerous regions subdivided further (e.g., Northeast Arctic vs. Norwegian coast). Considerable effort has been made to measure the extent to which these stocks represent biological populations and/or genetic demes; the answer remains equivocal. Early studies of hemoglobin protein markers found significant frequency differences among geographic locations (Cross & Payne, 1978; Frydenberg, Møller, Nævdal, & Sick, 1965; Gjøsæter, Jørstad, Nævdal, & Thorkildsen, 1992; Møller, 1968; Sick, 1965a,b), although selection and temperature differences have since explained many of these results (Jamieson & Birley, 1989; Mork & Sundnes, 1985). Trans‐Atlantic differences are evident in protein loci (Jamieson, 1967; Mork, Ryman, Ståhl, Utter, & Sundnes, 1985), mitochondrial cytochrome *b* (Carr & Marshall, 1991a; Sigurgíslason & Árnason, 2003), and nuclear DNA markers (Pogson, Mesa, & Boutilier, 1995), although results at local scales remain ambiguous. Extensive mtDNA analyses have found little or no structure in waters off the coasts of eastern North America (Carr, Snellen, Howse, & Wroblewski, 1995), Iceland (Árnason, Pálsson, & Arason, 1992; Árnason, Petersen, Kristinsson, Sigurgíslason, & Pálsson, 2000), the Faroe Islands (Sigurgíslason & Árnason, 2003), Norway (Árnason & Pálsson, 1996), and the Baltic Sea (Árnason, Petersen, & Pálsson, 1998). In contrast, microsatellite studies suggest range‐wide isolation by distance (O'Leary, Coughlan, Dillane, McCarthy, & Cross, 2007), and significant structure in waters off North America (e.g., Flemish cap, Bentzen, Taggart, Ruzzante, & Cook, 1996; inshore vs. offshore, Ruzzante, Taggart, Cook, & Goddard, 1996; Arctic populations, Hardie, Gillett, & Hutchings, 2006), Norway (Knutsen, Jorde, André, & Stenseth, 2003), and the North Sea (Hutchinson, Carvalho, & Rogers, 2001). Furthermore, genome scans of nuclear SNP markers have suggested the presence of local adaptation and temperature gradients at both large and small scales (Árnason & Halldórsdóttir, 2015; Berg et al., 2016; Bradbury et al., 2010, 2013; Nielsen et al., 2009).

**Table 1:** Sample locations, collection date, NAFO divisions (NAFO), sample size (*n*), number of haplotypes (h), haplotype diversity (H_d_), nucleotide diversity (π), and assignment to Bayesian cluster 1 or 2 for 153 complete mitochondrial genomes among 14 sampling locations of Atlantic cod

	Location	Collection date	NAFO	*n*	h	H_d_	π	1	2
NOR	Norwegian Coast off Tromsö	July 1989	n/a	7	7	1.00	0.0026	1	6
BLT	Baltic Sea at Sopot, Poland	April/May 2004	n/a	10	10	1.00	0.0027	3	7
QAS	Lake Qasigialiminiq, Baffin Island	November 2014	n/a	18	7	0.79	0.0020	6	12
LBH	Labrador—Hopedale	September 2013	2H	12	12	1.00	0.0028	4	8
LBM	Labrador—Makkovik	September 2013	2H	14	14	1.00	0.0016	1	13
LBP	Labrador—Postville	September 2014	2H	14	14	1.00	0.0018	0	14
HAW	Hawke Channel	June 1994	2J	10	10	1.00	0.0024	3	7
GIL	Gilbert Bay	June/July 1996	2J (inshore)	2	2	1.00	n/a	0	2
RAN	Random Island	June 1994	3L (inshore)	11	11	1.00	0.0021	3	8
NGB	North Cape of the Grand Banks	June 1994	3L	13	13	1.00	0.0017	1	12
FLM	Flemish Cap	July 1993	3M	11	11	1.00	0.0018	1	10
ESS	Eastern Scotian Shelf	July 1994	4W	11	11	1.00	0.0017	3	8
FSS	Fundy—Scotian Shelf	July 1994	4X	10	10	1.00	0.0023	2	8
GEO	Georges Bank	February 1995	5Ze	10	10	1.00	0.0022	2	8
Total				153	142	0.997	0.0023	30	123

Recently, complete mitochondrial genome sequences (usually 16–17 kbp among vertebrate species) have proven useful in clarifying phylogenetic relationships among species (Arnason et al., 2008; Cooper et al., 2001; Coulson, Marshall, Pepin, & Carr, 2006; Horai, Hayasaka, Kondo, Tsugane, & Takahata, 1995; Inoue, Miya, Tsukamoto, & Nishida, 2001; Miya, Kawaguchi, & Nishida, 2001; Miya et al., 2003). Following extensive use in human population and evolutionary genetics (Horai et al., 1995; Ingman, Kaessmann, Pääbo, & Gyllensten, 2000; Mishmar et al., 2003; Pope, Carr, Smith, & Marshall, 2011; Tanaka et al., 2004), increasing use has been made in intraspecific studies of fish (Carr & Marshall, 2008a; Feutry et al., 2014; Teacher, André, Merilä, & Wheat, 2012) and other mammals (Carr et al., 2015; Knaus, Cronn, Liston, Pilgrim, & Schwartz, 2011; Stone et al., 2010).

We present here a mitogenomic analysis of population genetic structure in Atlantic cod. Carr and Marshall (2008a) identified a much older time scale of population divergence than previously suspected, based on 32 mitogenomes from four populations from north and south of the Laurentian Channel, the offshore seamount Flemish Cap, and off Norway, as well as paraphyly among short (0.3–0.4 kbp) cytochrome *b* genotypes within six major mitogenomic lineages. We expand the data to a total of 153 mitogenomes by inclusion of 10 new populations from north and south of the Laurentian Channel in the Northwest Atlantic, the Baltic Sea, and a landlocked Arctic fjord at Lake Qasigialiminiq, Baffin Island (see Table S1).

## 2 MATERIALS AND METHODS

### 2.1 Sample collection and population units

A total of 153 Atlantic cod from 14 sampling locations were examined (Tables 1 and S1). Samples were collected by Fisheries and Oceans Canada from eight sampling locations in the Northwest Atlantic and by commercial trawlers from the Norwegian coast between July 1989 and February 1995 (Figure 2, Table 1). The Baltic Sea samples were collected by individuals at the Sopot Institute for Oceanology (Sopot, Poland) in April and May 2004. The Labrador and Baffin Island samples were collected by local fishermen between September 2013 and November 2014 (Table 1; see Acknowledgments). The 32 partial mtDNA genomes from Carr and Marshall (2008a; GenBank Accession Numbers EU877710‐EU877741; NOR, HAW, NGB, and FLM) are included here, with the addition of the complete control region sequence (see below).

Sampling locations in the Northwest Atlantic are designated by NAFO numbered divisions and lettered subdivisions (Table 1). Various combinations of these are designated as stocks for management. Samples from Hopedale, Makkovik, and Postville (LBH, LBM, and LBP; 2H) are grouped as Northern Labrador. Samples from Hawke Channel, inshore Gilbert Bay, inshore Random Island, and the North Cape of the Grand Banks (HAW, GIL, RAN, and NGB) are grouped as Northern cod (2J3KL). Samples from the Northern and Southern Scotian shelf (ESS and FSS; 4W and 4X) and Georges Bank (GEO; 5Z) are collectively “South” of the St. Lawrence, in contrast to 2H and 2J3KL as “North” of the St. Lawrence. The Flemish Cap (FLM; 3M) is a bathymetrically distinct offshore seamount that has been hypothesized as genetically distinct (Cross & Payne, 1978). Samples from the Barents Sea at Tromsö (NOR), the Baltic Sea at Sopot (BLT), and the landlocked fjord Lake Qasigialiminiq on Baffin Island, Nunavut (QAS) were each treated as ungrouped.

### 2.2 DNA extraction, amplification, and sequencing

DNA was extracted from heart tissue with the Qiagen DNeasy Blood and Tissue Kit according to the manufacturer's protocol (Qiagen, Hilden, Germany). The complete mitochondrial genome of 89 fish was amplified in a series of 23 or 24 standard PCR fragments ranging from 534 to 1,345 bp, with an average overlap of 169 bp (88–273 bp). Primers were originally designed for use with Atlantic cod (Coulson et al., 2006) or as part of this study. The control regions for the 32 partial mitogenomes from Carr and Marshall (2008a) were amplified using the primers g20F with g20‐2R1 and g20‐2F1 with g20R. PCRs were carried out using Qiagen PCR kits following the manufacturer's protocol with the following modifications: 2.0 mM MgCl_2_, 0.4 μM forward and reverse primer, and 40 extension cycles. Primer details and annealing temperatures are given in Table S2.

Five of the samples (3 LBH, 1 NGB, and 1 RAN) were also amplified in a series of overlapping long‐range PCR fragments ranging from 2,876 to 5,741 bp, with an average overlap of 406 bp (77–846 bp). The primer pairs used were g01F/g05R1, g05aF/g07bR, g07bF/g10R, g11F/g14R, and g14F/g20R (g05R1: CGA GTA AAC GGC GAG ACT TGA AAG G, g05aF: CCT ATG CCC TTT CCT GTA GCT GAT C, see Table S2, for all other primer sequences). Long‐range PCRs were carried out using the Qiagen LongRange PCR kit according to the manufacturer's protocol with the following modifications: 1.5 mM MgCl_2_, 0.4 μM forward and reverse primer, and 1.5 μl dimethyl sulfoxide. The thermal cycling program was as follows: an initial cycle of 2 min at 92°C; 10 cycles of 10 s at 92°C, 15 s at 58°C, 8 min at 68°C; 20 cycles of 15 s at 92°C, 30 s at 58°C, 8–17 min at 68°C (time increased by 20 s per cycle); and a final extension at 68°C for 7 min.

All PCRs were performed in an Eppendorf Mastercycler epGradient S thermocycler (Eppendorf, Hamburg, Germany) and then visualized under UV light on a 1% agarose gel with 0.3 μg/ml ethidium bromide. PCR products were purified with an Exo‐SAP clean‐up procedure; this consisted of incubation at 37°C for 15 min with 0.1 U exonuclease I (USB) and 0.1 U shrimp alkaline phosphatase (USB), followed by denaturation at 80°C for 15 min. Purified PCR products were sent to Genome Quebec (McGill University, Québec, Canada) for Sanger sequencing. Standard PCR products were sequenced in one or both directions; for fragments with extensive overlap, or where sequences showed no ambiguities, a single sequencing primer (either forward or reverse) was used. Long‐range PCR products were sequenced with a series of forward and reverse internal primers.

Thirty‐six samples were sequenced with the Affymetrix GeneChip^®^ CustomSeq^®^ resequencing multispecies microarray (the “ArkChip” Carr, Duggan, & Marshall, 2009). The samples were sequenced as part of two‐ and four‐species experiments, together with Atlantic wolffish (*Anarhichas lupus*; Duggan, 2007; Lait, 2016; Lait & Carr, under review), harp seals (*Pagophilus groenlandicus*; Carr et al., 2015), and Newfoundland caribou (*Rangifer tarandus*; Wilkerson, 2010; Wilkerson, Mahoney, & Carr, in press). For each sample, LR PCR products were amplified as above and pooled in equimolar quantities, fragmented, labeled, and sent to the Centre for Applied Genomics (Toronto, ON). They were then hybridized to the microarray chips and scanned with an Affymetrix GeneChip^®^ scanner (Duggan, 2007). Signal intensity data were exported to an Excel spreadsheet.

There were no differences between the two sequencing methods; samples did not cluster by method, and four samples were sequenced with both methods to ensure that there were no differences (see Table S1).

### 2.3 Data analyses

The microarray DNA data compare relative signal intensities of experimental‐to‐reference DNA binding and use a base‐calling algorithm based on a decision‐tree from empirical rules (Carr et al., 2008, 2009). For each individual, the data matrix consisted of two 4 × 16,576 arrays in which each of the four columns is one of the four possible bases (A, C, G, or T) at each of the 16,576 base positions; the two arrays are for the two complementary DNA strands. Experimental DNAs annealed to the reference sequence bind preferentially to the variant with the perfect match, which in >99% of cases is the same as the reference. The highest signal intensity is the presumptive call; the confidence in the call is estimated as a differential signal‐to‐noise ratio (dS/*N*), defined as the difference between the two highest signal intensities divided by the sum of all intensities. Empirically, calls made on both strands with dS/*N* ≥ 0.13 are considered “strong” and reliable, either in agreement with the reference sequence (invariant) or for a SNP variant, and calls with 0.10 ≤ dS/*N* < 0.13 are considered “weaker” but reliable if in agreement with the reference (Pope et al., 2011). Positions at which the two strand calls differ, or where dS/*N* < 0.10, were flagged with ambiguity codes for further analysis.

All sequences were aligned in Sequencher v4.9 (Gene Codes Corporation, Ann Arbor, MI). Variable sites were double‐checked across all genomes (microarray and/or chromatogram data). Positions at which ambiguous calls could not be reliably resolved were excluded from analyses. Sequences were aligned to the Atlantic cod reference sequence (Coulson et al., 2006), and the coding region annotations were confirmed against the partial Atlantic cod mitochondrial genome (Johansen & Bakke, 1996). Haplotypes were confirmed using TCS v1.2.1 (Clement, Posada, & Crandall, 2000) and Arlequin v3.5.1 (Excoffier & Lischer, 2010).

### 2.4 Genetic analyses

Default program values were used unless otherwise stated. Genetic diversity was measured with haplotype (H_d_) and nucleotide (π) diversity indices (Nei & Li, 1979; Nei & Tajima, 1981) calculated in DnaSP v5.10 (Librado & Rozas, 2009). An analysis of molecular variance (AMOVA) was performed in Arlequin v3.5.1 to allocate genetic variation within and among populations (100,000 permutations; Excoffier, Smouse, & Quattro, 1992; Excoffier & Lischer, 2010). A spatial analysis of molecular variance (SAMOVA) was used to partition populations based on both geographical and genetic data so as to maximize the among‐group variance (Φ_CT_), thus identifying any underlying structure and/or genetic barriers. The SAMOVA was calculated for *K* = 2–13 (100 iterations; Dupanloup, Schneider, & Excoffier, 2002).

Population pairwise genetic distances (Φ_ST_) for mtDNA genomes were calculated in Arlequin v3.5.1 (Excoffier & Lischer, 2010). A modified false discovery rate correction (Benjamini & Yekutieli, 2001) was applied to correct for multiple tests. Isolation by distance was tested with a Mantel test in Genepop v4.2 (10,000 permutations; Raymond & Rousset, 1995; Rousset, 2008). Straight‐line geographic distances were calculated from average GPS coordinates with the Geographic Distance Matrix Generator v1.2.3 (Ersts, 2015) as great circle distances; swimming distances were then calculated as the shortest marine distance between locations. These geographic distances were compared to linearized Φ_ST_ values (as genetic distances).

Phylogenetic relationships among haplotypes were analyzed with both Bayesian and neighbor‐joining (NJ) networks. The Bayesian network was assembled in MrBayes v3.2 (Huelsenbeck & Ronquist, 2001) with the generalized time reversible model, a gamma‐distributed rate variation, and allowance for invariable sites (GTR + Γ + I). The analysis was conducted as two simultaneous runs with eight chains for 5,000,000 generations, a 25% burn‐in, final PSRF > 0.999, ESS > 1,000, and standard deviation of split frequencies < 0.01. The NJ analysis was performed in PAUP* v4.10 (Swofford, 2003) based on the absolute number of nucleotide differences (10,000 bootstrap replications). The mitogenome of the congeneric Pacific cod *Gadus macrocephalus* (GenBank Accession Number AP017650) was used to root both analyses. Analyses rooted with Alaska pollock (*Gadus chalcogrammus*) as outgroup produce substantially the same result.

Clustering analysis was run with Bayesian Analysis of Population Structure (BAPS) v6 (Corander, Marttinen, Siren, & Tang, 2008). The analysis used the linked loci option, the codon model of linkage, and variable *K* (*K* ≤ 13; see Corander & Tang, 2007). The optimal *K* was determined based on the log marginal likelihood of the best‐visited partitions. An unrooted statistical parsimony network was constructed in TCS v1.21 with a 90% connection limit (Clement et al., 2000) to visualize the relationship between haplotypes. All connections were confirmed by visual examination. A principal coordinate analysis (PCoA) was carried out in GenAlEx v6.5 (Peakall & Smouse, 2006, 2012) on individual samples and on population pairwise differences.

### 2.5 Estimates of divergence and coalescent times

Divergence times were estimated from the Bayesian tree, based on the strict clock model in MrBayes v3.2 (Huelsenbeck & Ronquist, 2001; Ronquist & Huelsenbeck, 2003), and with the constant population model in BEAST v2.3 (Bouckaert et al., 2014). The MrBayes analysis was run as described above with uniform branch lengths. The BEAST analysis was run using a strict clock and the Hasegawa, Kishino, and Yano (HKY) model with gamma‐distributed rate variation and allowing for invariable sites (HKY + Γ + I). The models were run for 5,000,000 steps with a 25% burn‐in and sampled every 10,000 steps. All ESS parameters were > 1,000. The trees were calibrated with a normal distribution on the assumption that the Atlantic cod separated from the congeneric Pacific cod *G. macrocephalus* ca. 3.8 million years ago during the last opening of the Bering Strait (Coulson et al., 2006; Grant & Ståhl, 1988; Vermeij, 1991). The observed mean genetic distance of 0.039 substitutions per site then gives an estimated divergence rate of 1.03 × 10^−8^ substitutions/site/year, and a temporal interval of 5,844 years between substitutions. These values are similar to those calculated by Carr and Marshall (2008a) for the 15,655‐bp cod genome without the control region, and only slightly higher than that calculated for the Alaska pollock (Carr & Marshall, 2008b) and *Homo* (Achilli et al., 2004). Similar results are obtained with Alaska pollock (*Gadus chalcogrammus*) as the outgroup (Lait, 2016). Estimates of time since expansion were calculated in DnaSP v5.10 (Librado & Rozas, 2009) using pairwise mismatches and τ = 2μt (Rogers & Harpending, 1992).

### 2.6 Monte Carlo randomization test of phylogeographic structure

We used the Monte Carlo method described by Carr et al. (2015) to determine the degree to which the observed phylogeographic distribution of clades among sampling locations shows a significantly nonrandom fit to a priori models of geographic structure, as compared to the random distribution expected for such models. We grouped various sets of the 14 populations into four partitions, each comprising one or more populations, with a total count of N genomes, where the partitions comprised contiguous populations separated according to an a priori phylogeographic hypothesis. We tested three main models (I, I, and III) with two to six variants each (see Table 2). These were selected to explore (I) pan‐Atlantic differentiation, (II) trans‐Atlantic differentiation, and (III) structure within NAFO divisions in the Northwest Atlantic. Model I includes all genomes, with or without QAS. Model II considers four disjunct populations, including those in Carr and Marshall (2008a). Model III considers contiguous NAFO divisions in the Northwest Atlantic, variously with or without QAS or FLM.

**Table 2:** Monte Carlo tests of structured versus random phylogeographic distribution

	Description	*n*	Partition				L	C%	*p*
Ia	All genomes, aggregated	153	NOR + BLT	North + QAS	FLM	South	49	0.65	**
Ib	All genomes, aggregated, no QAS	135	NOR + BLT	North	FLM	South	48	1.05	*
IIa	Partition from Carr and Marshall (2008a)	41	NOR	HAW	NGB	FLM	17	0.27	**
IIb	NOR vs. BLT vs. NWA extremes	39	NOR	BLT	LBH	GEO	16	0.18	**
IIc	NOR vs. BLT vs. trans‐Laurentian	82	NOR	BLT	2J3KL	South	32	0.95	**
IIIa	NWA: no QAS or FLM	105	‐	2H	2J3KL	South	49	48.42	ns
IIIb	NWA: no FLM	123	QAS	2H	2J3KL	South	52	0.00	***
IIIc	NWA: no QAS	116	FLM	2H	2J3KL	South	59	43.73	ns
IIId	NWA: QAS vs. FLM vs. North	134	FLM	QAS + 2H	2J3KL	South	61	12.02	ns
IIIe	NWA: QAS vs. FLM vs. Middle	134	QAS	2H + 2J3KL	FLM	South	39	0.00	***
IIIf	NOR + BLT vs. NWA (no QAS or FLM)	122	NOR + BLT	2H	2J3KL	South	49	0.00	***

NWA, Northwest Atlantic; ns, not significant.

*p < .05; **p < .01; ***p < .001.

Composition of the partitions are indicated as 2H = LBH + LBM + LBP, 2J3KL = HAW + RAN + NGB, North = 2H + 2J3KL, and South = ESS + FSS + GEO. The length of the observed tree (L) and the % cumulative frequency of the L‐inclusive tail among 10,000 random trees (C%) are given. Refer to (Carr et al., 2015) for details of how L and C% are calculated.

## 3 RESULTS

### 3.1 Complete mitochondrial sequences

Complete mitogenomic sequences were obtained for 121 Atlantic cod from 14 locations (GenBank Accession Numbers KX266969–KX267089). These were combined with the 32 known partial mitogenome sequences with the addition of the 921‐bp control region sequence to each (GenBank Accession Numbers KX432219–KX432250). Results are reported for the combined set of 153 complete mitogenomes (16,576 bp in length based on one repeat unit, see below). A total of 887 variable sites were identified: 372 were parsimony informative (*sensu* Nei, 1987) and 515 were singletons. There were 779 transitions, 102 transversions, and six single‐base insertion‐deletion SNPs (five in the control region and one in the intergenic region between tRNA Thr and tRNA Pro). At 36 positions, SNP variants were three‐ or fourfold degenerate (*n* = 34 and 2, respectively). Of 706 coding region SNPs, 531 (75.2%) were third‐position transitions and 34 (4.8%) were first position transversions for alternative Leu codons. There were no frameshift or truncation mutations. The codon positions and substitution type of 87 SNPs imply amino acid substitutions, including three threefold variants (Leu ↔ Val ↔ Met, Leu ↔ Met ↔ Val, and Gly ↔ Ala ↔ Val).

Tandem repeats were found at two positions: a 40‐bp repeat in the control region (positions 15,696–15,735) and a 29‐bp repeat in the intergenic region between tRNA Thr and tRNA Pro (positions 15,577–15,605). Individual Atlantic cod from the Northeast Atlantic and adjacent waters have previously been shown to have one to six copies of the 40‐bp tandem repeat (Árnason & Rand, 1992; Johansen & Bakke, 1996; Johansen, Guddal, & Johansen, 1990; Kijewska, Burzyński, & Wenne, 2009). However, the 29‐bp repeat, found in three individuals from both sides of the Atlantic basin, has not been previously identified. Patterns of variation in the repeat units will be discussed elsewhere (L. A. Lait & S. M. Carr, in prep).

### 3.2 Population structure

A total of 142 distinct mitogenome sequences were identified. Of these, 139 were unique to single individuals, and three were found in two, five, and seven fish from Lake Qasigialiminiq. Population sample sizes ranged from 7 to 18 (Table 1), except for GIL (*n* = 2), which is excluded from all population comparisons below. Nucleotide diversity (π) within populations ranged from 0.0016 to 0.0028 (Table 1) with the lowest and highest values in the geographically adjacent LBM and LBH, respectively, the latter reflecting the basal position of two of the LBH samples. The AMOVA assigned 90.2% of the variation within populations (Φ_ST_ = .0978, *p* < .001). The SAMOVA identified the presence of *K* = 2 groups (QAS vs. all others; Φ_CT_ = .241, *p* = .065). With *K* = 3, BLT was separated from the others (Φ_CT_ = .217, *p* = .016), and at *K* = 4, NOR was separated (Φ_CT_ = .199, *p* = .003). Pairwise Φ_ST_ values (Table 3a) showed that the two European populations were significantly differentiated from most of the North American populations (five and six of 11 pairwise comparisons for NOR and BLT, respectively, after correction for multiple tests). QAS was significantly differentiated from all populations except BLT (*p* = .024). None of the other Northwest Atlantic comparisons were significant. The QAS sample comprised genomes in two unrelated haplogroups (see below); repetition of the analysis with these haplogroups separated as QAS1 and QAS2 identified them as significantly different from all other populations, including each other (*p* < .001). There was no significant correlation between genetic and geographic distance including trans‐Atlantic comparisons (*p* = .088, *R* ^2^ = .005), nor among populations in the Northwest Atlantic taken separately (*p* = .783, *R* ^2^ = .020).

**Table 3:** Population pairwise Φ_ST_ values (below diagonal) and corresponding *p*‐values (above diagonal) based on 100,172 permutations for complete mitogenomes from (a) 13 populations of Atlantic cod, and (b) NAFO stock classifications

(a)	NOR	BLT	QAS	LBH	LBM	LBP	HAW	RAN	NGB	FLM	ESS	FSS	GEO
NOR	–	ns	**	ns	**	**	ns	ns	***	*	*	**	ns
BLT	.082	–	*	*	***	***	*	**	***	ns	**	**	*
QAS	.253	.153	–	***	***	***	**	***	***	**	***	***	**
LBH	.029	.082	.236	–	*	ns	ns	ns	ns	ns	ns	ns	ns
LBM	.085	.153	.315	.042	–	ns	ns	ns	*	ns	*	*	ns
LBP	.062	.140	.301	.001	.000	–	ns	ns	ns	ns	ns	ns	ns
HAW	.029	.086	.263	.000	.040	.004	–	ns	ns	ns	ns	ns	ns
RAN	.033	.121	.276	.000	.020	.000	.000	–	ns	ns	ns	ns	ns
NGB	.120	.180	.340	.014	.046	.008	.030	.013	–	ns	ns	ns	ns
FLM	.064	.075	.245	.009	.023	.000	.020	.005	.013	–	ns	ns	ns
ESS	.047	.114	.292	.000	.044	.000	.000	.000	.019	.000	–	ns	ns
FSS	.065	.105	.271	.000	.060	.015	.012	.006	.005	.000	.000	–	ns
GEO	.033	.093	.258	.000	.036	.000	.000	.000	.009	.000	.000	.000	–

(b)	NOR	BLT	QAS	2H	2J3KL	3M	4W	4X	5Ze
NOR	–	ns	**	*	*	*	*	**	ns
BLT	.082	–	*	***	***	ns	**	**	*
QAS	.253	.153	–	***	***	**	***	***	**
2H	.061	.133	.279	–	ns	ns	ns	ns	ns
2J3KL	.071	.148	.295	.003	–	ns	ns	ns	ns
3M	.064	.075	.245	.000	.005	–	ns	ns	ns
4W	.047	.114	.292	.000	.000	.000	–	ns	ns
4X	.065	.105	.271	.023	.007	.000	.000	–	ns
5Ze	.033	.093	.258	.003	.000	.000	.000	.000	–

Refer to Table 1 for locations.

(a) p_crit_ = .0101; ns = not significant, *p < .05; **p < .01; ***p < .001.

(b) p_crit_ = .012; ns = not significant, *p < .05; **p < .01; ***p < .001.

**Figure 3:**
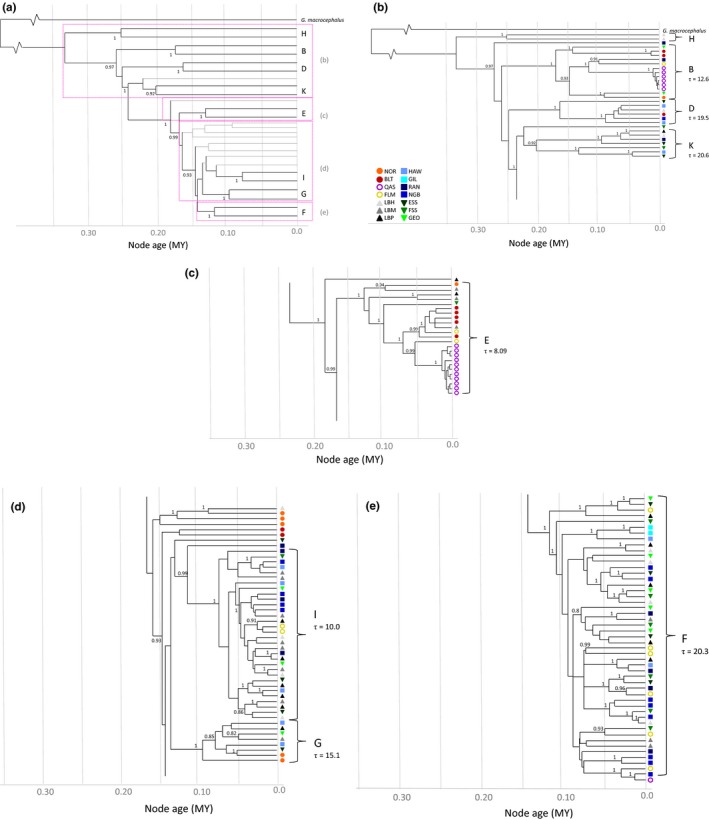
Clock‐calibrated Bayesian analysis of complete mitogenomes from 153 Atlantic cod. The horizontal axis shows time since separation in million years. Posterior probabilities ≥ .8 are given. (a) Simplified Bayesian analyses showing the eight major haplogroups B–K. Gray lines lead to mitogenomes that do not fall into one of the eight major haplogroups (labeled) with high confidence, and (b–e) a detailed view of the Bayesian analysis for each of the eight major haplogroups

The Bayesian analysis identified 63 branches with posterior probabilities > .95 (Figure 3). These include the six haplogroups previously identified (B, D, E, F, G, and I; Carr & Marshall, 2008a), an additional two haplogroups (H and K), and numerous subhaplogroups within the larger groups. Haplogroup H is basal to all other groups. The neighbor‐joining analysis identified 47 branches with >70% bootstrap support (Figure S1), which includes as clusters the same eight haplogroups found in the Bayesian analysis. The distribution of haplogroups across populations was significantly heterogeneous (Χ^2^ = 214.5, *df* = 117, *p* = .004): NOR, BLT, and QAS were found almost exclusively in haplogroups B, E, and G (Figure 3b–d), and no European samples and only a single QAS sample were found in the two most abundant haplogroups (F and I; Figure 3d,e). If NOR, BLT, and QAS were excluded, the distribution of haplogroups across populations was not significantly different from random (Χ^2^ = 106.7, *df* = 90, *p* = .908).

**Figure 4:**
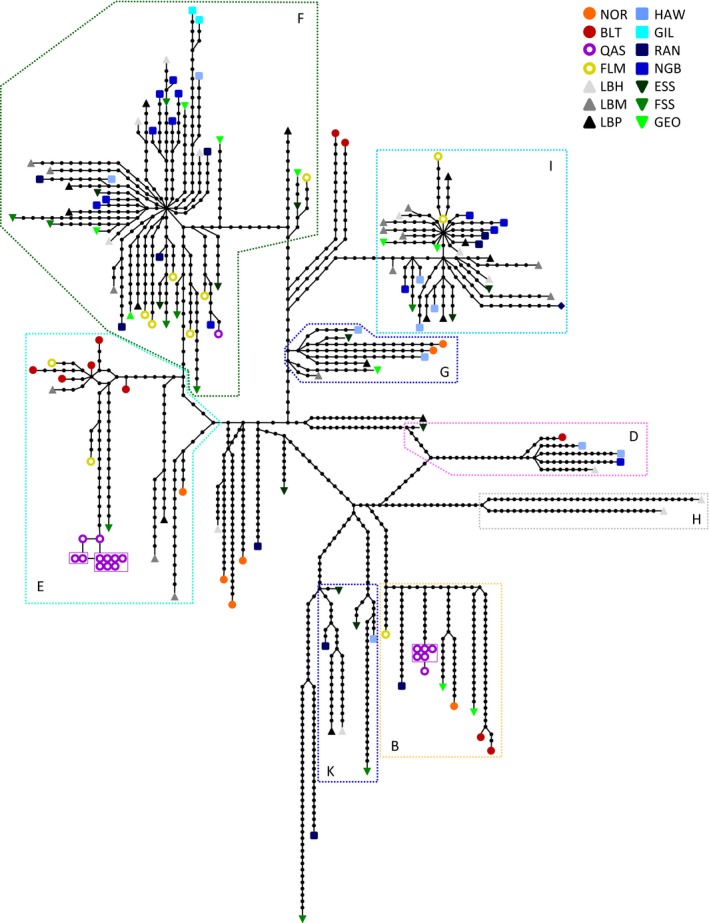
Statistical parsimony network of complete mitogenomes for 153 Atlantic cod. Each symbol represents an individual, the black dots are inferred haplotypes, and each connection represents one nucleotide change. Shared haplotypes are encased by a box, and the dashed boxes correspond to the haplogroups identified in Figure 2. Refer to Table 1 for locations

**Figure 5:**
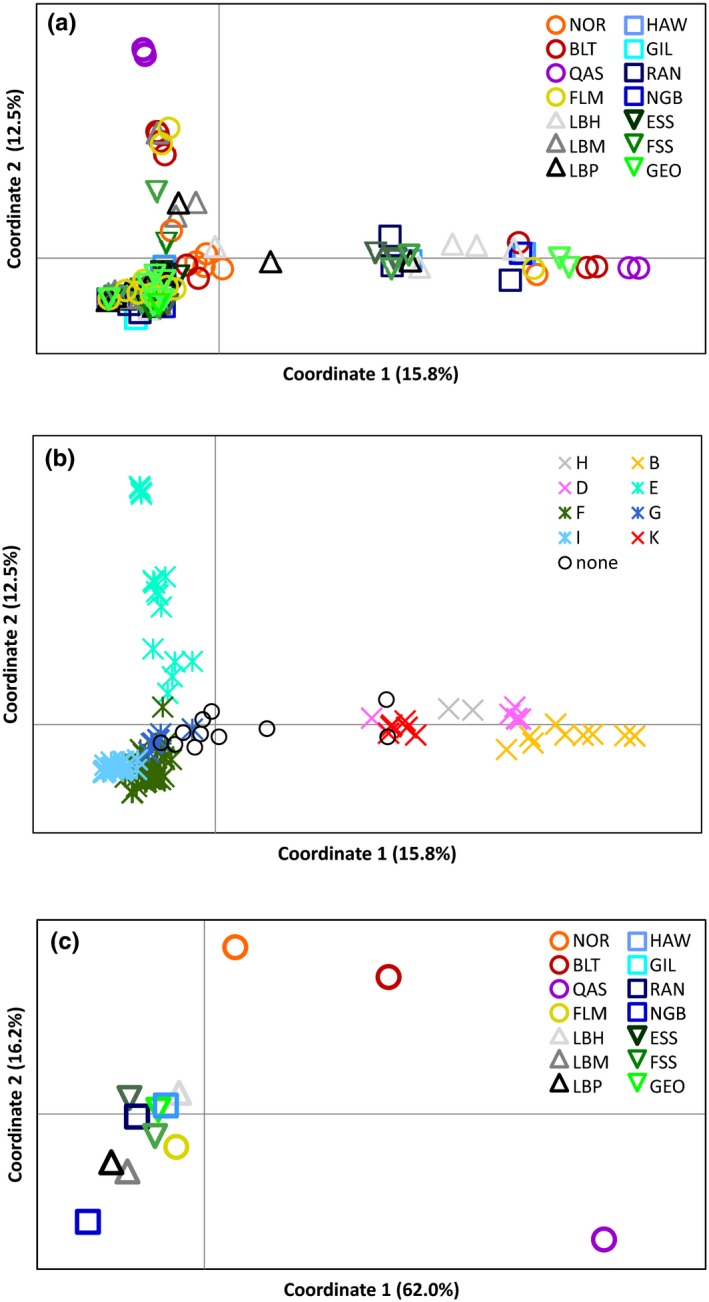
PCoA based on (a) 153 individual mtDNA sequences colored by population, (b) 153 individual mtDNA sequences colored by haplogroup where “none” refers to samples that did not fall into one of the eight major haplogroups, and (c) the population Φ_ST_ values for 13 populations (excluding GIL). Populations are color‐coded as per Figure 2. Refer to Table 1 for locations

Bayesian clustering analysis (Table 1) identified two groups, one corresponding to the four most basal haplogroups (H, B, D, and K; Group 1) and the other the four more recent haplogroups (E, F, G, and I; Group 2). The three highest log maximum likelihood values were −14,637 (*K* = 2), −15,080 (*K* = 3), and −15,831 (*K* = 4); the probability of two clusters was unity. The distribution of populations between the two groups was not significantly heterogeneous (Χ^2^ = 13.13, *df* = 13, *p* = .438). The statistical parsimony network (Figure 4) showed extensive variation in Atlantic cod and identified the same groups found in the Bayesian and NJ analyses. The QAS samples comprised two essentially monomorphic haplotypes in two disjunct haplogroups (B and E), with one sample in haplogroup F. Half of the BLT samples clustered together in a nonexclusive subhaplogroup within E, and the others in B, D, and basally within the FGI superhaplogroup. The BLT and QAS mitogenomes in haplogroup E are sister haplogroups. NOR mitogenomes were not closely grouped with those of any other population (16–86 substitutions), nor with each other (29–61 substitutions). Variation in the two most abundant haplogroups (F and I) arose suddenly, as evidenced by the star‐like pattern, and they did not include any Northeast Atlantic (NOR or BLT) mitogenomes. The PCoA analysis (Figure 5a) identified the same two groups as the Bayesian clustering analysis, with additional substructuring in group 2. Coordinates I, II, and III explained 15.8%, 12.5%, and 8.8% of the variation, respectively. The more basal haplogroups (H, B, D, and K) separated from the younger haplogroups (E, F, G, and I) along axis I, and the Northeast Atlantic haplogroup (E) separated from the others along axis 2 (Figure 5b). The PCoA on the population Φ_ST_ values (Figure 5c) again supported limited structure among the Northwest Atlantic populations, with the exception of QAS (62.0% coordinate 1, 16.2% coordinate 2, and 10.1% coordinate 3). QAS separated from all other populations along axis I, NOR along axis II, and BLT along axes I and II.

The Monte Carlo dispersal models, matrices, and results for eight partitions are given in Table 2. Most of the *a priori* partitions of haplogroups among populations were significantly shorter than random, including the all‐inclusive partitions with or without QAS (*p* = .0065 and .0105, respectively), and the four‐population trans‐Atlantic partition corresponding to Carr and Marshall (2008a; *p* = .0027). Most Northwest Atlantic‐only models were not significantly shorter than random, either with QAS included or excluded (*p* = .1202 and *p* = .4373, respectively). Alternatives that excluded either FLM alone or together with QAS remained highly significant (*p* < .0001 in either case). This indicates that the diphyletic composition of QAS did not unduly lengthen random trees, and that trans‐Atlantic populations were significantly differentiated.

### 3.3 Estimated species coalescent and haplogroup divergence times

Based on the estimated divergence rate of 1.03 × 10^−8^ substitutions/site/year, the most recent common ancestor of all Atlantic cod examined dates to 333 kya (95% highest posterior density (HPD) = 215–435 kya), and the superhaplogroup containing the more recent expansions in the Northwest Atlantic (FGI) dates to 145 kya (HPD = 75–170 kya). The coalescence times of the eight major haplogroups range from 47 to 120 kya, all well before the last glacial maximum (Figure 3). The pairwise mismatch distribution suggests sudden expansion rather than a constant population size, with τ = 23.39 corresponding to an expansion at 137 kya. The expansion of the Northwest Atlantic FGI superhaplogroup dates to 110 kya.

### 3.4 NAFO divisions as *a priori* hypothesis of stock structure

An AMOVA with the NAFO stock divisions allocated 89.7% of the variation within populations and 10.3% among populations (Φ_ST_ = .1031, *p* < .001). Among the 36 pairwise Φ_ST_ population comparisons, 12 were significant, all of which involved NOR, BLT, and/or QAS. QAS was significantly different from all other populations, except BLT, and none of the among‐North Atlantic stock comparisons were significant (Table 3b).

## 4 DISCUSSION

### 4.1 Population genomic structure

Atlantic cod comprise eight major haplogroups, as supported by Bayesian, distance, parsimony, and principal coordinates analyses (Figures 3, 4, 5 and S1), including six previously identified haplogroups (Carr & Marshall, 2008a), a sister haplogroup (K) to the EFGI superhaplogroup, and a new basal haplogroup from NAFO 2H (H). There is trans‐Atlantic differentiation, high diversity in Norwegian waters and the Baltic Sea, a polyphyletic Arctic lake isolate, and limited differentiation among populations in the Northwest Atlantic.

Extensive gene flow and admixture is suggested by nonsignificant Φ_ST_ values among all but the Qasigialiminiq and Northeast Atlantic sampling locations (Table 3a), and the absence of isolation by distance among the Northwest Atlantic samples. The separation of trans‐Atlantic populations is incomplete—in many comparisons (e.g., among the geographic extremes LBH, FLM, HAW, and GEO), there were no differences between populations from either side of the Atlantic, whereas in others there was strong isolation (e.g., LBP, LBM, NGB, and FSS; Table 3a). This pattern is similar to that seen in the congeneric Pacific cod *Gadus macrocephalus*, where a combination of short mtDNA sequences (ND2 and CYTB) and microsatellites showed significant differences between trans‐Pacific populations, and no distinction among geographically proximal locations (Canino, Spies, Cunningham, Hauser, & Grant, 2010). In this case, the authors suggested multiple glacial refugia on either side of the North Pacific Ocean with significant admixture following deglaciation.

In the Monte Carlo analysis, all five models that examined trans‐Atlantic partitions (Models I and II, see Table 2) indicated significant structure (*p* = .002–.010). This includes the complete set of 153 genomes aggregated broadly as [NOR + BLT], FLM, North, and South of the Laurentian Channel (Ia). (The influence of QAS in Model Ia is discussed below.) It also includes partitions that separate NOR and BLT. Model IIa corresponds to the partition in Carr and Marshall (2008a; NOR, HAW, NGB, and FLM), which was tested for a difference in tree length between a Northwest versus Northeast Atlantic rooting. Here, the Monte Carlo partition falls in the 0.27% tail of random expectation, which indicates structure based on trans‐Atlantic differentiation. In contrast, Models IIIa and IIIc test the geographic extremes of structure in the offshore Northwest Atlantic considered on its own, and neither departs from random (*p* > .4).

The present results are broadly consistent with past studies on cod based on microsatellite data and/or short mtDNA sequences, except for paraphyly in the latter (see Carr & Marshall, 2008a for a discussion on paraphyly in cod sequences). Based on one set of microsatellite markers, Ruzzante et al. (1996) claimed significant differentiation among some offshore and inshore‐spawning aggregations in NAFO 2J3KL, but Carr and Crutcher (1998) noted this was found only in some partitions and was dependent on a single marker. With the same set of markers, Bentzen et al. (1996) found heterogeneity between Labrador (North) and Grand Banks (South) samples, and also suggested that the Flemish Cap and Scotian Shelf populations were significantly differentiated. Hardie et al. (2006), with a different set of microsatellite markers, found differences among the three landlocked Baffin Island populations, but no differences among populations south of the Laurentian Channel. O'Leary et al. (2007) claimed strong evidence of isolation by distance, but included only one Northwest Atlantic population (from the Scotian Shelf) whose closest geographic neighbor was from Greenland. Analyses with short mtDNA cytochrome *b* sequences (307–401 bp) alone consistently suggest an absence of genetic differentiation within regions and trans‐Atlantic differences in haplotype frequencies (Árnason, 2004; Árnason & Pálsson, 1996; Árnason et al., 1992, 1998, 2000; Carr & Crutcher, 1998; Carr & Marshall, 1991a,b; Carr et al., 1995; Hardie et al., 2006).

Whereas the majority of cod populations are migratory between inshore feeding grounds and offshore spawning locations (Lear, 1984; Templeman, 1979), a historic hypothesis is the existence of “bay cod” that remain more or less permanently inshore in deepwater bays that surround the island of Newfoundland (Carr et al., 1995). However, mitogenomes from inshore‐spawning fish near Random Island in Trinity Bay and at Gilbert Bay in southern Labrador (RAN and GIL) are not differentiated from the nearest offshore populations (NGB and HAW) and are no less diverse as might be expected in an isolated population (cf. QAS).

BAPS analysis separated out the QAS and BLT populations from all others, which reflects their co‐occurrence in the B and E haplogroups. Although both Baffin Island and the Baltic Sea basin were glaciated until ca. 5,000–8,000 years ago, their genetic patterns contrast sharply. This is likely due to differences in how they were recolonized and their subsequent effective population numbers. Lake Qasigialiminiq is physically small and has maintained a very small census population size; it shows an odd polyphyletic composition and reduced haplotype diversity. In contrast, the Baltic Sea has apparently maintained a very large population size that includes diverse basal lineages, and has maintained high diversity and no shared haplotypes.

### 4.2 Landlocked Arctic fjord population

The shared haplotypes seen in the landlocked fjord population at Lake Qasigialiminiq, at the southwest end of Cumberland Sound, Baffin Island, seem to reflect recent isolation and maintenance at small population size (500–1,000 individuals). Shared mitogenomic haplotypes are not seen in marine populations of Atlantic cod, and the presence of multiple distinct, private haplotypes within a single population is unprecedented in marine fish mitogenomic studies. The major haplogroups in Lake Qasigialiminiq are polyphyletic, and, by implication, of distinct temporal origins, although a single colonization event by a mixed population is also possible. The QAS fish assigned to haplogroup F is most closely related to an individual in NGB and is likely a more recent introduction from a lineage that is otherwise restricted to the Northwest Atlantic.

With respect to the Monte Carlo test, the QAS sample might be expected to introduce a somewhat artifactual distinction between the observed pattern of clustered identical or near‐identical haplotypes, versus a randomized distribution in which extradispersal events are necessarily required to account for random scatter. Put another way, as the two clusters in the B and E haplogroups and the singleton F mitogenome found in QAS require exactly three events in the observed tree, their accommodation in any random tree requires more. However, probabilities of the observed trees in Model I variants are similar with or without QAS (Ia vs. Ib: *p* = .0065 vs. .0105). In the various partitions of the Northwest Atlantic in Model III, the two variants that include QAS separately are highly structured (IIIb and e: *p* < .001), whereas variants that group it with NAFO 2H or exclude it altogether are not (IIIa, c, and d; *p* > .1; Table 2).

The identical QAS genomes occur in haplogroups that are either basal (B) or shared predominantly with BLT cod (E). This suggests a closer phylogeographic affinity with the east than with the west; this is supported by recent findings that the Greenland cod populations were colonized from Iceland (Therkildsen et al., 2013), and supports a westerly movement. Additional samples from the Northeast and mid‐Atlantic would help to clarify this. The calibrated Bayesian tree puts the origin of diversity in either haplogroup at <20 kya (Figure 3). This probably overcorrects pairwise differences of zero or one SNP, recalling that the mean interval between fixations is ca. 5,000 years. This agrees temporally but contrasts biogeographically with the hypothesis that the three Arctic lakes (or at least Lake Qasigialiminiq) were colonized from the south by Northwest Atlantic cod ca. 5,000–8,000 years ago (Hardie et al., 2006). The individual from haplogroup F indicates a third invasion, consistent with the latter hypothesis. That the lake at present includes descendants of at least three distinct lineages suggests that either a single source population was highly variable and the lake has maintained variation despite opportunities for a founder effect and subsequent drift, or that there were at least three invasion events. Atlantic cod have been absent from the Davis Strait farther north than northern Labrador since the last glacial maximum, making a recent trans‐Atlantic event unlikely. However, if water temperatures in the Arctic Ocean and the Davis Strait were several degrees higher from 3,000 to 6,000 years ago (Aitken & Gilbert, 1989), such movements may have been possible.

Based on partial cytochrome *b* fragments, Hardie et al. (2006) assigned all cod sampled in the three Baffin Island lakes to haplotypes “*A*,” “*E*,” or “*G”* (*sensu* Carr et al., 1995), where “*A”* is the most common, widespread haplotype in Atlantic cod. Of 18 QAS samples, our data for the homologous region would assign 11 and seven mitogenomes as identical within haplotypes “*E”* and “*A*,” respectively. Mitogenomic analysis assigns the former to a tight cluster within haplogroup E, whereas six of the “*A*” samples are instead assigned to the more basal haplogroup B and one to the younger haplogroup F (Figures 3 and 4; Figure S1). Carr and Marshall (2008a) discusses the implications of paraphyly in short versus complete mtDNA sequences; in brief, the single locus studies often fail to detect structure and cryptic lineages, which can result in misleading or incomplete conclusions.

Paraphyly of short mtDNA sequences may also be suspected in other isolated populations of Atlantic cod. Zhivotovsky et al. (2016) describe the genetics of landlocked Atlantic cod in meromictic Lake Mogilnoye on Kildin Island (so‐called Kildin cod, *Gadus morhua kildinensis* Derjugin 1920) separated from the mainland by a narrow strait in the Barents Sea. Based on a 970 bp mtDNA cytochrome *b* sequence, Zelenina, Makeenko, Volkov, and Mugue (2016) identified 60 haplotypes among fish in Lake Mogilnoye, the Barents and Baltic Seas, and 26 fish from Carr and Marshall (2008a) from the Northwest Atlantic, including 12 distinct haplotypes from the latter. A shared haplotype was found in all 30 landlocked Kildin fish. This haplotype is also present in Barents and White Sea fish, as well as in the homologous segment of the mitogenome of a fish from HAW (Carr & Marshall, 2008a), and in the present data, it occurs in fish from BLT, LBH, HAW, and NGB. The latter, all found in haplogroup D, differ by 15–22 pairwise substitutions elsewhere in the mitogenome. Otherwise, analysis of the 60 eastern haplotypes and their equivalent regions in the present data shows extensive paraphyly of nominally identical haplotypes. Examination of complete mitogenomes might reveal polyphyletic origins of Kildin cod, with origins in both the Barents and White Seas.

### 4.3 Glacial refugia

The Pleistocene consisted of a series of ice ages interspersed with shorter warm periods (Hewitt, 2000, 2004). During the most recent Wisconsinan (Nearctic) or Würm (Palaearctic) glaciations, ice sheets covered much of the Northern Hemisphere. Sea levels were as much as 120 m lower than today, exposing continental shelves that became covered with ice (Mitrovica, 2003; Pielou, 1991). Haplogroup expansion in Atlantic cod (Figure 3) coincides with the interglacial and warming periods experienced during the Wisconsinan (ca. 110–12 kya) and Illinoian (ca. 200–130 kya) glaciations (Gibbard & van Kolfschoten, 2004). Origins of the most abundant Northwest Atlantic haplogroups occurred ca. 113 kya (F) and 145 kya (FGI), which correspond to the Sangamonian interglacial. Many of the other separations are estimated to have occurred at some point in the Illinoian glaciation; there were at least two warming periods ca. 250 and 300 kya (Gibbard & van Kolfschoten, 2004; Wright, 2000), and much of the early divergence dates to these warmer periods, or even prior to the Illinoian.

The patterns of variation seen in Atlantic cod suggest distinctive Northeast and Northwest Atlantic cod populations. Pairwise comparisons show that the Norway and Baltic Sea populations are significantly differentiated from the Northwest Atlantic populations in 75% of comparisons (Table 3). Both the AMOVA and the PCoA (Figure 5) were also heavily influenced by these two populations. As seen in previous studies, despite a lack of fixed differences there appears to be significant trans‐Atlantic differentiation. Bradbury et al. (2013) identified consistent differences between Northwest Atlantic and European samples with neutral and non‐neutral markers. The magnitude of the separation suggests that it predates the most recent glacial cycle, and may be a relic of the Illinoian glaciation or earlier. The lack of fixed allelic differences and geographical structuring among marine cod populations suggests that recent admixture and gene flow may be confounding older interglacial patterns.

During the last glacial maximum, suitable habitat occurred in the Northeast Atlantic, in the western Atlantic south of the ice sheets, and may have occurred along the continental shelves of Atlantic Canada including the Flemish Cap, the Grand Banks, and in the Davis Strait between Baffin Island and western Greenland (Bigg et al., 2008; Maggs et al., 2008). Nucleotide diversity (Table 1) is highest in the samples from the Barents and Baltic Seas, which may represent source populations. This contrasts with the “Codmother” hypothesis (Carr & Marshall, 2008a), which suggested a Northwest Atlantic origin of the current variation. This hypothesis relied on the occurrence of basal haplogroup D only in the Northwest Atlantic; it has now been found as well in the Barents and Baltic Seas. With the discovery of the more basal haplogroups B and H (the former including Baltic Sea cod), H has replaced D as the oldest haplogroup, which is confined at this time to northern Labrador. Populations on the other side of the Atlantic basin remain undersampled. Although the Baltic Sea was covered in ice prior to ca. 7,000 years ago, fish may have persisted in an adjacent, brackish refuge and subsequently recolonized large areas of the southwestern and southeastern Baltic, such that no sharp decline in effective population size is evident (Poćwierz‐Kotus et al., 2015; Poulsen, Nielsen, Schierup, Loeschcke, & Grønkjær, 2005). Zelenina et al. (2016) argue for distal distribution of White Sea cod cytochrome *b* haplotypes with respect to those in the Barents Sea. The trans‐Atlantic differences seen in Atlantic cod, and the absence of NOR and BLT samples from the younger F and I haplogroups, suggest that there may have also been expansion from one or more glacial refugia in the Northwest Atlantic. Traditional candidates are an emergent Flemish Cap, the Grand Banks, or a more southerly trans‐Laurentian refugium. Cross and Payne (1978) from allozyme genotypes, and Carr and Marshall (1991b) from short cytochrome *b* sequences, suggested that shared genotypes between the Flemish Cap and Barents Sea samples indicated trans‐Atlantic dispersal by way of middle latitudes. Based on complete mitogenomes, the Flemish Cap sample does not differ significantly from any Northwest Atlantic or trans‐Laurentian population (Φ_ST_ = .000–.023, *p* > .15; Table 3a). These data suggest that the Flemish Cap likely either did not act as a refugium for Atlantic cod, or that any refugial distinctiveness has since been eliminated by gene flow. It is worth noting that FLM also does not differ significantly from BLT (Φ_ST_ = .075, *p* > .05), and that although Flemish Cap fish occur predominantly in haplogroup F, they are also present in every other haplogroup except the two smallest (D and H). Likewise, the NGB data do not support the Grand Banks as a possible refugium.

### 4.4 Shared mitogenomes in other species

Every marine Atlantic cod had a unique DNA sequence, as has been seen in the highly variable Alaska pollock (*Gadus chalcogrammus*; Carr & Marshall, 2008b), Atlantic herring (*Clupea harengus*; Teacher et al., 2012), and harp seal (*Pagophilus groenlandicus*; Carr et al., 2015). However, the landlocked population had mitogenomes shared among multiple individuals and unique to that population. In contrast, Atlantic wolffish (*Anarhichas lupus*; Lait, 2016; Lait & Carr, under review), green turtle (*Chelonia mydas*; Shamblin et al., 2012), and speartooth shark (*Glyphis glyphis*; Feutry et al., 2014) all include mitogenomes shared among individuals that are widely dispersed. The latter suggests recent origin of these lineages followed by dispersal, whereas the limitation of identical cod to a confined space is suggestive of founder effect and/or local drift on a scale of a few thousand years. Study of the mtDNA genome composition of the other fjord lakes would be of interest.

## 5 CONCLUSIONS

The current picture of genetic diversity in Atlantic Cod is based on sequence analysis of mitochondrial DNA (Carr & Marshall, 2008a) and nuclear SNP markers (Bradbury et al., 2010, 2013; Nielsen et al., 2009). Due to its wide‐scale distribution and pelagic ecology, Atlantic cod have been predicted to have limited population structure over small geographical scales, and only weak structure in trans‐Atlantic comparisons. Although this was found in early studies of short mtDNA sequences, a greater degree of population structure has been suggested based on some nuclear microsatellites at local scales, and with most markers at larger scales.

The mitogenomic data indicate little or no stock differentiation within the commercial range of the Northwest Atlantic. The clock‐calibrated Bayesian network shows multiple haplogroup expansions ca. 120–100 kya, around the Sangamonian interglacial/Wisconsinan glacial boundary, that comprise the most common haplotypes found in North America. This suggests contemporaneous recovery from one or possibly more western Atlantic refugia. However, diversity measures and the predominance of basal haplogroups support the eastern Atlantic and adjacent waters as an origin and ultimate source population. Haplogroup H is anomalous in this respect, but may be an artifact of geographic undersampling, as is apparent in retrospect for haplogroup D in Carr and Marshall (2008a). As the pattern of variation does not indicate a common geographic location of origin, it is likely that historically separated populations have undergone secondary admixture. Identity of mitogenomes from Lake Qasigialiminiq in the west, and perhaps Lake Mogilnoe in the east, shows the expected patterns in isolated populations founded since the LGM. Additional samples from across the range, particularly from mid‐ and eastern Atlantic and adjacent waters, may help to resolve uncertainties regarding refugial identification and colonization routes. The high level of variation seen in the cod mitogenomic sequences, including the trans‐Atlantic differences and isolation of Arctic fjord populations on Baffin Island, shows that mitogenomics has sufficient power to resolve these questions.

## DATA ACCESSIBILITY

DNA sequences: GenBank accession numbers KX266969–KX267089 (whole mitogenomes); KX432219–KX432250 (control regions). Primer sequences: uploaded as online supporting information (Table S1).

## CONFLICTS OF INTEREST

None declared.

## AUTHOR CONTRIBUTIONS

SMC and HDM conceived the ideas; HDM and LAL collected the samples; LAL and HDM collected the data; LAL and SMC analyzed the data; SMC and LAL funded the project, and LAL led the writing with contributions from all authors.
