# Supplementary material for: Phylogeographic mitogenomics of Atlantic cod Gadus morhua: Variation in and among trans‐Atlantic, trans‐Laurentian, Northern cod, and landlocked fjord populations
Source: Ecol Evol. 2018 Jun 1;8(13):6420–37. doi: 10.1002/ece3.3873 (PMC6053584; doi:10.1002/ece3.3873)
Supplement: Supplementary file 1 [file ECE3-8-6420-s001.docx]

**Supplementary Information**

**Table S1.** Method of sequencing for 153 Atlantic cod samples from 14 sampling locations. The "C&M" samples are those from Carr & Marshall ([2008a](#_ENREF_23)), the "Arkchip" samples were sequenced with the Arkchip resequencing microarray, and the Sanger samples were sequenced with standard Sanger sequencing of overlapping fragments.

|  | C&M | Arkchip | Sanger | Total |
| --- | --- | --- | --- | --- |
| NOR | 6 |  | 1 | **7** |
| BLT |  | 10 |  | **10** |
| QAS |  |  | 18 | **18** |
| LBH |  |  | 12 | **12** |
| LBM |  |  | 14 | **14** |
| LBP |  |  | 14 | **14** |
| HAW | 8 |  | 2 | **10** |
| GIL |  |  | 2 | **2** |
| RAN |  |  | 11 | **11** |
| NGB | 9 | 4^1^ |  | **13** |
| FLM | 9 | 2 |  | **11** |
| ESS |  | 10 | 1 | **11** |
| FSS |  |  | 10 | **10** |
| GEO |  | 10 |  | **10** |
| Total | **32** | **36** | **85** | **153** |

^1^ Four NGB samples were sequenced with both the Arkchip and Sanger. They are only listed once here.

**Table S2.** Primers used for PCR amplification of Atlantic cod. Primer pairs are given; where possible g01F was used with g02R (T_A_ = 52°C) and g09aF with g09bR (T_A_ = 52°C). Annealing temperatures (T_A_; °C), location of 5'-most base of the primer (loc), and primer sources (ref) are given.

| Name | Sequence (5' to 3') | T_A_ | loc | ref |
| --- | --- | --- | --- | --- |
| g01F | CTGAAGATATTAGGATGGACCCTAG | 49 | 29 | 1 |
| g01R | CTAGTCCCTACTTACTGCTAAATCC | 49 | 871 | 1 |
| g02F | CCAAAAACGTCAGGTCGAGGTGTAG | 55 | 742 | 1 |
| g02R | CTATTCATTTCACAGGCAACCAGCT | 55 | 1,490 | 1 |
| g03F | ACCCCGAAACTGAGCGAGCTACTCC | 52 | 1,351 | 1 |
| g03R | TAAGCCCTCGTGATGCCATTCATAC | 52 | 2,150 | 1 |
| g04F | TTTACCAAAAACATCGCCTCTTG | 52 | 1,995 | 1 |
| g04R | TGAACCTCTGTAGAAAGGGCTTAGG | 52 | 2,815 | 1 |
| g05F | GGAGTAATCCAGGTCAGTTTCTATCTATG | 52 | 2,600 | 1 |
| g05aR | AAAGTGGTGTAGTGGAAGCAGTAGG | 52 | 3,944 | 2 |
| g06F | GGTTAAAGTCCCTTCAACTCCTTAG | 52 | 3,856 | 1 |
| g06aR | GGTAGTGTCCTGCAGATCTTATCAG | 52 | 5,153 | 2 |
| g07aF | CTACCTTACCTCTCGCTATTTCAGC | 55 | 4,978 | 2 |
| g07aR | CTACACCAGAGGATGCTAAAAGGAG | 55 | 5,798 | 2 |
| g07bF | ATAATTGGAGGCTTTGGGAACTGAC | 55 | 5,663 | 2 |
| g07bR | TGTTAAGCCCCCGACTGTAAAGAGG | 55 | 6,505 | 2 |
| g08F | ATGGGTATAGTCTGAGCTATGATGG | 48 | 6,254 | 1 |
| g08R | TAACCCACAATTCTGCCTTGACAAG | 48 | 7,121 | 1 |
| g09aF | ACATTCGAGGAACCCGCATTCGTTC | 52 | 6,953 | 2 |
| g09aR | GAGGTCTTCGTAATCGGTSTSCTCG | 52 | 7,499 | 2 |
| g09bF | GCCCTTCCATCATTACGAATTCTTTATC | 52 | 7,380 | 2 |
| g09bR | ATTAAGGGTGGTTGGGAGTCACCTGCTT | 52 | 7,914 | 2 |
| g10F | TCCCGGAGTTTTCTACGGACAATG | 47 | 7,715 | 1 |
| g10R | AGAGGGCGAATGAATAAACTAATTG | 47 | 8,539 | 1 |
| g11F | TAGCAACTGTCCTTATCGGCATACG | 47 | 8,407 | 1 |
| g11R | TAATACTGTGGTGAGCTCAGGTTAC | 47 | 9,212 | 1 |
| g12aF | ATTATCCGAGAGGGGACCTTTCAGG | 55 | 8,939 | 2 |
| g12aR | CGTAGGGAGATAGCTTTTCGTAGTC | 55 | 9,728 | 2 |
| g12bF | CAACAGTTATCCTTATTGCCTCAGC | 55 | 9,627 | 2 |
| g12bR | GTTAAGAGCAGTCCATCCGGTATCC | 55 | 10,483 | 2 |
| g13F | CTTTCTCCGCTTGTGAAGCAAG | 47 | 10,225 | 1 |
| g13R | CAATTAGAGATTTCAGGTCAGTTTG | 47 | 11,183 | 1 |
| g14F | CTGTTGCAGGCTCAATAGTTCTTGC | 52 | 10,995 | 1 |
| g14R | TTCGAGGGAGCCTTGGGGTCTAACC | 52 | 11,841 | 1 |
| g15F | TAACCAAGACATTAGATTGTGATTC | 48 | 11,715 | 1 |
| g15R | TGGTAGTCATGGGTGAAGTCCAAAC | 48 | 12,634 | 1 |
| g16F | GGTGATGACACGGCCGAGCAGATG | 57 | 12,396 | 1 |
| g16R | AATAATTGCATCTTTGGAGAAGAAGC | 57 | 13,120 | 1 |
| g17F | ATTCATAGCCTAAACGATGAACAAG | 52 | 12,971 | 1 |
| g17R | GTCGTTTTTCATATCATTAGTCCTG | 52 | 14,316 | 1 |
| g18F | GCTACTAAGACCAGTCCTAAAGCAG | 48 | 14,164 | 1 |
| g18R | CTGTGGGATTATTTGAGCCTGTTTC | 48 | 14,971 | 1 |
| g19F | GAGGAGGTTTCTCAGTAGATAATGC | 48 | 14,837 | 1 |
| g19R | GTTTAATTTAGAATTCTAGCTTTGG | 48 | 15,690 | 1 |
| g20F | GAATGAAACTGCCCTAGTAGCTCAG | 47 | 15,475 | 1 |
| g20-2R1 | TGGACCTGAAGCTAGGCAGAATAGC | 47 | 16,098 | 2 |
| g20-2F1 | GTAAACATAACCGGACTTTCCTTGC | 49 | 15,981 | 2 |
| g20R | GGCAGGACATTAAGGGCATTCTCAC | 49 | 160 | 1 |

1 ([Coulson *et al.* 2006](#_ENREF_37))

2 This study

**Figure S1.** Neighbour-joining analysis of complete mitogenomes from 153 Atlantic cod. Bootstrap values > 70% are given (10,000 replicates). Refer to Table 1 for locations.
